# Risk of chronic periodontitis in patients with obstructive sleep apnea in Korea: a nationwide retrospective cohort study

**DOI:** 10.4178/epih.e2023032

**Published:** 2023-03-06

**Authors:** Seon-Rye Kim, Minkook Son, Yu-Rin Kim

**Affiliations:** 1 Department of Healthcare Management, Youngsan University, Yangsan, Korea; 2 Department of Physiology, Dong-A University College of Medicine, Busan, Korea; 3 Department of Dental Hygiene, Silla University, Busan, Korea

**Keywords:** Chronic periodontitis, Oral health, Obstructive sleep apnea

## Abstract

**OBJECTIVES:**

The aim of this study was to determine whether the development of chronic periodontitis is more likely among patients who have been newly diagnosed with obstructive sleep apnea (OSA) through an analysis of representative data from the general population.

**METHODS:**

A nationwide, population-based, retrospective cohort study was conducted using patient records from the Korean National Health Insurance Service database. For the period 2004-2019, patient data were categorized into 2 groups: a diagnosis of OSA (747 subjects) and no diagnosis of OSA (1,494 subjects). Subsequently, 1:2 propensity score matching was performed to ensure the homogeneity of the 2 groups. To analyze the risk of incident chronic periodontitis, a Cox proportional-hazards model was used to calculate hazard ratios (HRs) with 95% confidence intervals (CIs).

**RESULTS:**

In the Kaplan-Meier curve, the disease-free probability was significantly lower in the OSA group than in the non-OSA group (p for log-rank test=0.001). The crude HR for the association between OSA and chronic periodontitis was 1.29 (95% CI, 1.16 to 1.43). The multivariable-adjusted HR was calculated at 1.28 (95% CI, 1.15 to 1.42).

**CONCLUSIONS:**

This study confirmed a relationship between OSA and chronic periodontitis. Therefore, OSA patients require oral care to prevent the progression of chronic periodontitis from mild to severe.

## GRAPHICAL ABSTRACT

[Fig f3-epih-45-e2023032]

## INTRODUCTION

Sleep breathing disorders are collectively referred to as obstructive sleep apnea (OSA), central sleep apnea, and sleep-related hypoventilation syndromes. Among them, OSA accounts for the majority of sleep-breathing disorders. Its incidence increases in male after middle age and in female after menopause. The prevalence of OSA in male in their 30s and 40s is about 10%, versus only about 3% for female in the same age range; however, after age 50, the prevalence of OSA increases by about 2 times in male and about 3 times in female [1]. In Western Europe and the United States, the prevalence of OSA is 24.0-49.7% in male and 9.0-23.4% in female, and the incidence is very high [2,3].

Multiple research studies on OSA have already confirmed its associations with comorbidities, including cardiovascular disease, metabolic disease, and stroke [4]. In addition, OSA was reported to be associated with chronic periodontitis due to mouth breathing during snoring, which is a prominent symptom of OSA [5]. Mouth breathing, which leads to a dry mouth, is associated with OSA [6]. It has been reported that dryness due to prolonged oral breathing increases the risk of periodontitis by promoting bacterial colonization and accumulation [7,8]. As another factor, patients with OSA have periodic recurrent hypoxemia and reoxygenation damage. Alternation between hypoxemia and reoxygenation can elevate levels of proinflammatory cytokines and free radicals, leading to oxidative stress and inflammatory responses [9]. Similarly, patients with periodontitis have increased levels of inflammatory cytokines, which destroy periodontal tissue [10,11].

The inflammatory biomarkers associated with OSA and chronic periodontitis include interleukin (IL)-1, IL-6, IL-8, tumor necrosis factor-α (TNF-α), and high-sensitivity C-reactive protein (hs-CRP). Consequently, proinflammatory immune responses may result in a bidirectional link between these 2 diseases [12]. Accordingly, many studies have reported a significant association between OSA and chronic periodontitis [13-17]. In a review of 10 studies with a cumulative total of 30,994 participants, periodontitis was reported to be directly related to OSA, but not related to severe OSA, emphasizing the need for further studies [18]. However, other researchers have conducted conflicting analyses that found no association between OSA and the prevalence of periodontitis or severe periodontitis [19-21].

Therefore, the relationship between OSA and chronic periodontitis remains a matter of debate, and domestic studies related to this study pair are lacking. The only large-scale domestic study related to OSA and periodontitis was a cross-sectional study of genomic epidemiology in Korea, with 687 participants from 2009 to 2010. According to that study, OSA was significantly associated with periodontitis, pocket depth, and clinically attached gingival levels, but the researchers noted that further studies would be needed to clarify the causal relationship between the 2 conditions [5]. No previous studies have used the Korean National Health Insurance Service (KNHIS) database to analyze the relationship between OSA and chronic periodontitis. Therefore, to address this gap in the literature, the purpose of this study was to analyze the relationship between OSA and chronic periodontal disease using the KNHIS database. The study was conducted with the goal of obtaining basic data to support clinicians in advising OSA patients of the importance of oral health by pointing out their risk of chronic periodontitis.

## MATERIALS AND METHODS

### Definition of variables

#### Definition of OSA and non-OSA subjects

The subject population for the study was derived from the KNHIS database [22] using records dated between January 1, 2002 and December 31, 2019. To identify subjects with a first-time OSA diagnosis, the World Health Organization’s International Statistical Classification of Diseases and Related Health Problems 10th revision (ICD-10) was used to extract individuals diagnosed with code G47.3 (n=5,551) [23]. In addition, to increase the accuracy of OSA subject selection, individuals who visited the hospital at least once within 1 year of their initial diagnosis of OSA were extracted (n=3,016). After that, a washout period from January 1, 2002 to December 31, 2003 was established, and individuals diagnosed with OSA during that period were excluded (n=2,629). Individuals with a diagnosis of multiple sclerosis (G35), neuromyelitis optica (G36), optic neuritis (H46), giant cell arteritis (M31.5, M31.6), or other systemic connective tissue diseases (M35.3) were also excluded (n=22). Individuals diagnosed with OSA or diagnosed with chronic periodontitis before the index date were excluded from this study (n=1,706). We excluded individuals with missing data (n=154). The final total of the study population was 747 subjects.

The non-OSA (control) group was defined as subjects who were not diagnosed with OSA during the same period. To secure the homogeneity of the OSA and non-OSA groups, the index year, sex, income level, underlying disease, and Charlson comorbidity index (CCI) were matched at a 1:2 propensity score (n=2,241). In addition, we performed sensitivity analyses with 1:3 (n=2,988) and 1:4 (n=3,735) propensity score matching.

**Figure 1.**
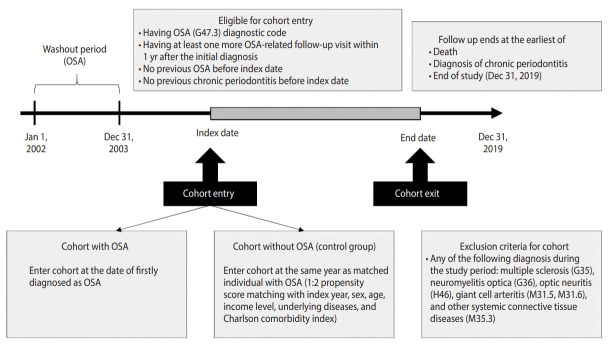
Study population flow. OSA, obstructive sleep apnea.

Follow-up was terminated at the time of death, diagnosis with chronic periodontitis, or the study’s endpoint of December 31, 2019 (Figure 1, Supplementary Material 1).

#### Covariates

The covariates included age, sex, income level (quartile groups), hypertension (I10-I15), diabetes (E10-E14), dyslipidemia (E78), heart disease (I20-I25), cerebrovascular disease (I60-I69), and CCI (Supplementary Material 1). The CCI was calculated from underlying conditions during the follow-up period, including myocardial infarction, congestive heart failure, peripheral vascular disease, cerebrovascular disease, dementia, chronic pulmonary disease, connective tissue disease, peptic ulcer, mild liver disease, diabetes with and without complications, paraplegia or hemiplegia, renal disease, any cancer (primary or metastatic), moderate or severe liver disease, and acquired immune deficiency syndrome [24]. The CCI may be ranked by weighting conditions from 1 to 6 according to disease type and severity with an evaluation range of 0-37 [25].

#### Definition of chronic periodontitis

Chronic periodontitis was defined using the ICD-10 diagnostic codes K051 and K053 [26], and the treatment codes for chronic periodontitis were specified as U2232, U2233, U2240, U1010, U4412, U4413, U1051, U1052, U1071, U1072, U1081, U1082, U1083 and UY101. We used this combination of diagnosis and treatment codes to identify chronic periodontitis (Supplementary Material 1).

### Statistical analysis

KNHIS data were analyzed using R version 3.6.0 (R Foundation for Statistical Computing, Vienna, Austria) and SAS version 9.4 (SAS Institute Inc., Cary, NC, USA). For baseline characteristics in the OSA and non-OSA groups, the Student t-test and the chi-square test were used to compare the variables for adjustment. A Kaplan-Meier curve was presented for chronic periodontitis risk analysis, and the log-rank test was performed. The incidence rate of chronic periodontitis was presented in units of 1,000 person-years for the total follow-up period of the OSA and non-OSA groups. To analyze the risk of chronic periodontitis, a Cox proportional-hazards model was constructed to calculate the hazard ratio (HR) and 95% confidence intervals (CIs). In order to ensure the reliability of the results, age, sex, income level, hypertension, dyslipidemia, and CCI were adjusted. A p-value < 0.05 was considered to indicate statistical significance.

### Ethics statement

The study was approved by the Human Subjects Ethics Board of Youngsan University (IRB No. YSUORB-202208-HR-118-02) and was conducted in accordance with the Helsinki Declaration of 1975, as revised in 2013.

## RESULTS

### Demographic characteristics in the study groups

The baseline characteristics for the subject population in this study are described in Table 1. Of the 2,241 total subjects, 747 had OSA and 1,494 did not. The average duration of follow-up was 5.1 years. The subjects in both groups were at least 65 years old, and there were more male than female. The fourth quartile of income predominated, while the fewest participants were found in the first quartile. As for the prevalence of underlying diseases, 48.9% of the non-OSA subjects had hypertension, compared to 48.1% of the OSA group. The corresponding rates were 8.9% and 11.1% for diabetes, 19.4% and 19.5% for dyslipidemia, 27.8% and 28.5% for heart disease, and 16.2% and 17.3% for cerebrovascular disease, respectively. The CCI was 2.9 in the non-OSA group and 3.0 in the OSA group. All underlying diseases except for hypertension were present at higher proportions in the OSA group than in the non-OSA group, but without statistically significant differences (p> 0.05). In contrast, chronic periodontitis was present in 63.1% of the non-OSA group and 72.7% of the OSA group, which constituted a significant between-group difference (p<0.001).

### Comparison of risks of cerebrovascular and cardiovascular diseases according to the severity of chronic periodontitis

**Figure 2.**
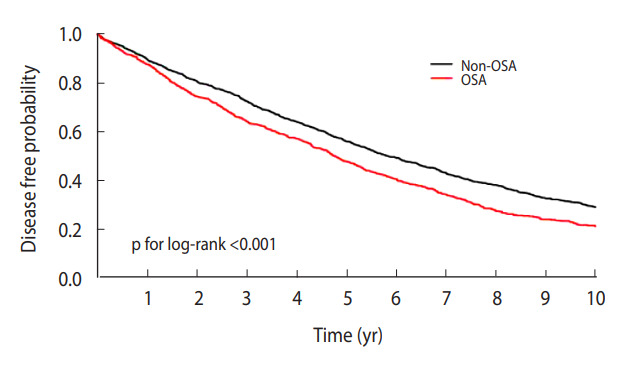
Kaplan-Meier curve for the association between obstructive sleep apnea (OSA) and chronic periodontitis.

The probability of disease-free status was prominently lower in the OSA group than in the non-OSA group on the Kaplan-Meier curve (log-rank test: p<0.001; Figure 2). OSA was significantly associated with chronic periodontitis in the crude and multivariable-adjusted Cox proportional hazard models. The crude HR for the association between OSA and chronic periodontitis was 1.29 (95% CI, 1.16 to 1.43). In the multivariable-adjusted HR, the value was 1.28 (95% CI, 1.15 to 1.42). In sensitivity analyses with 1:3 and 1:4 propensity score matching, the multivariable-adjusted HRs and 95% CIs were 1.23 (1.11 to 1.35) and 1.24 (1.12 to 1.36), respectively.

## DISCUSSION

The results of this nationwide, population-based cohort study showed that OSA was a risk factor for chronic periodontitis. This association remained significant even after adjusting for well-known confounding factors of chronic periodontitis including age, sex, income level, hypertension, diabetes, dyslipidemia, heart disease, cerebrovascular disease, and the CCI. This is the first large-scale study to demonstrate the incidence of chronic periodontitis in an OSA population and to analyze the effect of OSA on the occurrence of chronic periodontitis.

Previous studies have suggested an association between OSA and chronic periodontitis [13-17]. According to a cross-sectional study by Stazić et al. [16], stronger symptoms of sleep apnea were reported in OSA patients with severe periodontitis compared to those with either mild or no periodontitis. According to a meta-analysis of clinical data by Zhu et al. [15], both periodontal pocket depth and clinical attachment loss (CAL) were higher in the OSA group than in the control group, and gingival bleeding was also more common. In addition, an in vitro study confirmed an increase in Prevotella, a periodontal pathogen, in the salivary microbiome structure of OSA patients; this result indicates that OSA patients have a high risk of chronic periodontitis [13]. A study by Al Habashneh et al. [14] demonstrated that patients with a high risk of OSA had almost twice the risk of developing periodontitis compared to patients with a low risk of OSA. In the current study, chronic periodontitis occurred 1.28 times more frequently in the OSA group than in the non-OSA group. The reason for this difference is that, unlike previous studies that analyzed the risk of chronic periodontitis in OSA patients, our study compared the occurrence of chronic periodontitis between OSA and non-OSA groups.

Chronic periodontitis is an irreversible inflammatory disease that requires specialist care to maintain the support structure of teeth and limiting disease progression. The disease has several etiological factors and risk factors, including aging, sex, obesity, heredity, stress, pregnancy, nutrition, diabetes, and smoking. To exclude these factors from the current study, the OSA and non-OSA groups were matched through a 1:2 fixed ratio propensity score. For this reason, the number of selected subjects decreased, but the reliability of the results increased. In this 16-year study, there was no significant difference between the two groups in terms of age, sex, income level, or underlying diseases; however, the incidence of chronic periodontitis was 72.7% in the OSA group, which was significantly higher than in the non-OSA group. These results are related to the pathophysiological factors of OSA patients, as described above.

TNF-α, IL-6, and hs-CRP levels have been reported to be higher in OSA patients due to repeated events of hypoxia and apnea, and IL-6 and IL-33 have also been found to be significantly higher in severe OSA patients [27,28]. In addition, snoring in OSA patients causes both bad breath and dry mouth. When the self-purifying action of saliva is lost due to dry mouth, bacteria form colonies, and periodontitis occurs as a result [29]. From a behavioral point of view, OSA patients reported lower daily brushing habits, resulting in a higher plaque index [29].

Loke et al. [30] justified the association between OSA and periodontitis by confirming that periodontal pockets were deep and CAL was high in severe OSA patients. Gunaratnam et al. [31] later demonstrated in a clinical trial that systemic inflammation and metabolism improved when periodontitis was treated in OSA patients. Another population-based study reported a lower OSA rate with appropriate periodontal treatment in patients with severe periodontitis, emphasizing the importance of managing periodontitis in patients with OSA [17]. Therefore, OSA patients will need not only to improve their treatment for OSA, but also to change their habits (e.g., mouth breathing) that increase the incidence of periodontitis. Patients should also be well educated in effective tooth-brushing methods to form positive oral hygiene habits, and periodic scaling is required to prevent chronic periodontitis from progressing further. OSA patients would also benefit from counseling to relieve stress, which would require collaboration across various medical fields.

This cohort study has several important limitations. First, the sample size in this study was relatively small, and a cohort study design cannot demonstrate the causal relationship between OSA and periodontitis. Second, the health checkup data used in this study did not provide clinical indicators of moderate OSA and chronic periodontitis, so the relationships could not be evaluated in more detail. Therefore, additional clinical and experimental studies are needed to establish the underlying causal relationship. Nevertheless, this study is meaningful as the first study to confirm the relationship between OSA and chronic periodontitis in Korea. In addition, relatively strict inclusion criteria were established to exclude confounding factors, which is ideal for investigating possible influencing factors. In this study, OSA patients had a higher risk of chronic periodontitis than non-OSA patients. To decrease the incidence of OSA, oral care from dental professionals and active education to prevent periodontitis are needed.

In conclusion, according to this study’s results, the risk of chronic periodontitis was high in people with OSA. In order to manage chronic periodontitis in OSA patients, dental staff need to consider various aspects, including oral anatomical factors, psychological factors, and behavioral factors such as habit improvement. Thus, sufficient knowledge about the characteristics of OSA patients will make it possible to improve professional collaboration among various medical services.

## Figures and Tables

**Figure f3-epih-45-e2023032:**
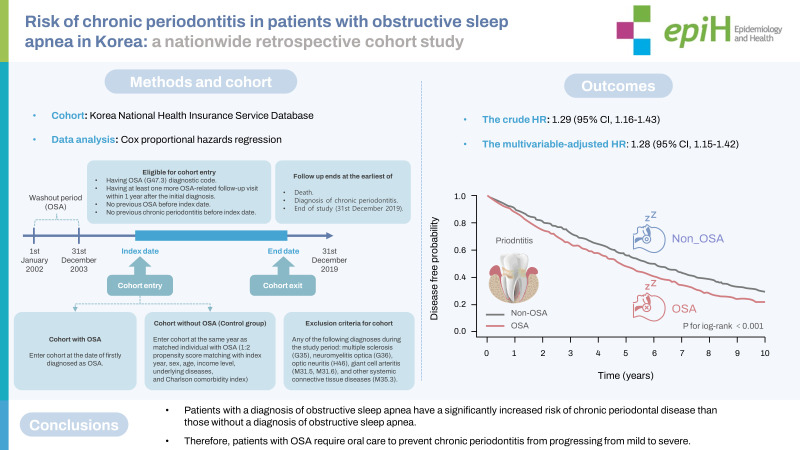


**Table 1.** Baseline characteristics of the study population

Characteristics		OSA (n=747)	Non-OSA (n=1,494)	p-value
Demographics				
	Age	56.1±7.6	56.2±7.8	0.72
	Sex			0.51
	Male	597 (79.9)	1,213 (81.2)	
	Female	150 (20.1)	281 (18.8)	
	Income level (quartile)			0.94
	1st	65 (8.7)	127 (8.5)	
	2nd	101 (13.5)	209 (14.0)	
	3rd	195 (26.1)	404 (27.0)	
	4th	386 (51.7)	754 (50.5)	
Underlying disease				
	Hypertension	359 (48.1)	730 (48.9)	0.75
	Diabetes	83 (11.1)	133 (8.9)	0.12
	Dyslipidemia	146 (19.5)	290 (19.4)	0.99
	Heart disease	213 (28.5)	416 (27.8)	0.78
	Cerebrovascular disease	129 (17.3)	242 (16.2)	0.56
	Charlson comorbidity index	3.0±2.6	2.9±2.5	0.44
Outcome				
	Chronic periodontitis	543 (72.7)	943 (63.1)	<0.001

Values are presented as number (%) or mean±standard deviation. OSA, obstructive sleep apnea.

## SUPPLEMENTARY MATERIALS

Supplementary Material 1. Definitions of diseases by disease classification codes and insurance claim codes
