# Supplementary material for: Risk of chronic periodontitis in patients with obstructive sleep apnea in Korea: a nationwide retrospective cohort study
Source: Epidemiol Health. 2023 Mar 6;45:e2023032. doi: 10.4178/epih.e2023032 (PMC10586923; doi:10.4178/epih.e2023032)
Supplement: Supplementary Material 1. — Definitions of diseases by disease classification codes and insurance claim codes [file epih-45-e2023032-Supplementary-1.docx]

**Supplementary Material 1. Definitions of diseases by disease classification codes and insurance claim codes**

| **Disease** | **ICD-10 codes** | **Claim codes** |
| --- | --- | --- |
| OSA | G47.3 | Outpatient ≥ 1 |
| Chronic periodontitis | K051, K053 | Dental procedures including U2232, U2233, U2240, U1010, U4412, U4413, U1051, U1052, U1071, U1072, U1081, U1082, U1083, UY101 |
| Hypertension | I10 – I15 | Outpatient ≥ 1 |
| Diabetes  Dyslipidemia  Heart disease  Cerebrovascular disease | E10 – E14  E78  I20 – I25  I60 – I69 | Outpatient ≥ 1  Outpatient ≥ 1  Outpatient ≥ 1  Outpatient ≥ 1 |

ICD-10, Tenth Revision of International Classification of Diseases
